# Biogenic Silica Nanoparticles Enhance *Beauveria bassiana* Bioavailability and Efficacy for Sustainable Pest Control

**DOI:** 10.1021/acsomega.5c11436

**Published:** 2026-05-13

**Authors:** Liliam Katsue Harada, Mariana Guilger-Casagrande, Tais Germano-Costa, Natália Bilesky-José, Ricardo Antonio Polanczyk, Kelly Cristina Gonçalves, Paulo Gonçalves da Silva, João Vitor Silva e Silva, Renato de Mello Prado, Leonardo F. Fraceto, Renata Lima

**Affiliations:** † Laboratory for Evaluation of the Bioactivity and Toxicology of Nanomaterials, 130242 University of Sorocaba (UNISO), Rod. Raposo Tavares km 92.5, 18023-000 Sorocaba, São Paulo, Brazil; ‡ Laboratory of Microbial Control of Pests, São Paulo State University (UNESP), Via de Acesso Paulo Donato Castellane s/n, 14884-900 Jaboticabal, São Paulo, Brazil; § Laboratory of Plant Nutrition, Department of Agricultural Production Sciences (Soil and Fertilizer Sector), São Paulo State University (UNESP), Via de Acesso Paulo Donato Castellane s/n, 14884-900 Jaboticabal, São Paulo, Brazil; ∥ Laboratory of Environmental Nanotechnology, São Paulo State University (UNESP), Av. Três de Março 511, 18087-180 Sorocaba, São Paulo, Brazil

## Abstract

Biogenic silicon dioxide nanoparticles (SiO_2_–NPs_bio) synthesized using *Fusarium oxysporum* were combined with *Beauveria bassiana* in a Pickering emulsion to enhance fungal bioavailability and insecticidal efficacy. The biogenic nanoparticles exhibited larger diameters (324 ± 3 nm) and lower ζ-potential (−23 ± 0.2 mV) than commercial SiO_2_–NPs, while showing no significant cytotoxicity in HaCat and 3T3 cell lines. Encapsulation markedly improved fungal performance, resulting in 100% mortality of *Spodoptera frugiperda*, *S. cosmioides*, and *Chrysodeixis includens*, and 86% mortality of *Rachiplusia nu*, significantly higher than isolated nanoparticles or fungus. The nanoformulation also protected *B. bassiana* against UV radiation and showed no phytotoxic effects. This study demonstrates, for the first time, a sustainable biogenic SiO_2_-based Pickering emulsion as an effective carrier for entomopathogenic fungi in pest control.

## Graphical abstract

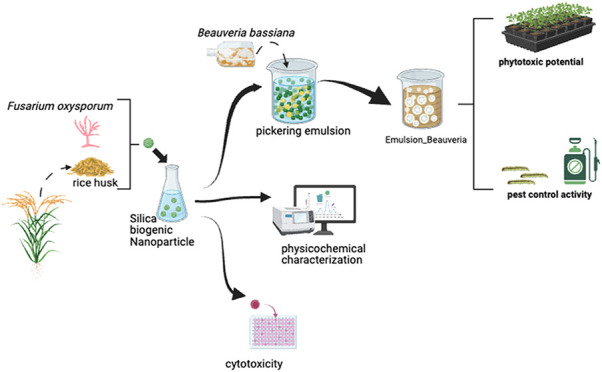

## Introduction

The use of new technologies to enhance crop yield with the lowest environmental impact is essential in the current agricultural scenario. In this context, the bioeconomy aligns with the Sustainable Development Goals (SDGs), which are essential for managing the sustainability of agricultural activities, enhancing quality, biological activity, and efficiency, while reducing environmental impacts.^1^ According to this premise, the use of micro- and nanotechnology solutions, such as the encapsulation of microorganisms and the biogenic synthesis of nanoparticles, is an alternative that protects the biological control agents from abiotic factors, optimizes their release, and stimulates their growth and reproduction.^2,3^

The green synthesis of silicon nanoparticles is a promising alternative due to its use of residues, which constitute an environmentally and economically resilient strategy.^4,5^ The nanoparticles are desirable as carrier materials due to their biocompatibility, stability, and low toxicity, as well as their attractive potential as nanofertilizers, due to the enhancement of plant growth, and nanopesticides, due to the insecticidal activity through the dissection of the insect cuticle.^6,7^ These aspects enable the formation of consortia with microorganisms for agricultural applications since the association of silicon nanoparticles with microorganisms may increase the efficiency of the microbial agents as well as improve soil fertility and nutrient uptake.^8^

In this way, the combination of conidia of entomopathogenic fungi, such as *Beauveria bassiana*, with silicon nanoparticles may contribute synergistically to pest control and act as a protective agent, enhancing the efficiency and reducing the effects of external factors and mechanical stress, which may have a direct impact on the bioavailability. *B. bassiana* is widely used against agricultural pests, which have caused considerable reductions in soybean yields.^3,9,10^ The efficiency of this fungus is attributed to the production of toxins, such as beauvericin and bassianolide, which act in the nervous system of the target insects, causing mortality.^11^

On the basis of the affirmation supported by predictive algorithms and high technological tools, that the biological control in integrated pest control management systems may confer a higher efficiency of pest management,^12^ this study aimed to perform the synthesis, characterization, and evaluation of the toxicity of silicon nanoparticles obtained from rice husk using the *Fusarium oxysporum* fungus, owing to its ability to promote the bioleaching of amorphous silica and its subsequent biotransformation into crystalline silica.^13^ In addition, a microemulsion system was developed for the combined encapsulation of the nanoparticles with *B. bassiana* conidia, using a Pickering emulsion as a sustainable alternative for pest control in agriculture.

In this context, biological control activity was associated with the process of synthesizing nanoparticles from agricultural waste. These points are interesting in an economic and environmental approach, which aligns with the 13th SDG (climate action), reducing carbon emissions from the burning of agricultural residues.^14,15^ In addition, nanomaterials have shown potential as plant growth promoters and triggers of plant tolerance against stress, acting as nanofertilizers and nanopesticides, which may improve agricultural yield and sustainability, contributing to the second SDG (zero hunger) and the 12th SDG (responsible consumption and production).^16–18^

## Results and Discussion

Figure 1 illustrates the performed activities since the synthesis of the biogenic silicon nanoparticles (SiO_2_–NPs_bio), their characterization, the production of the emulsion, the incorporation of microorganisms, and the evaluation of toxicity and bioactivity. Concurrently, the tests were performed with commercial nanoparticles (SiO_2_–NPs_com).

**Figure 1:**
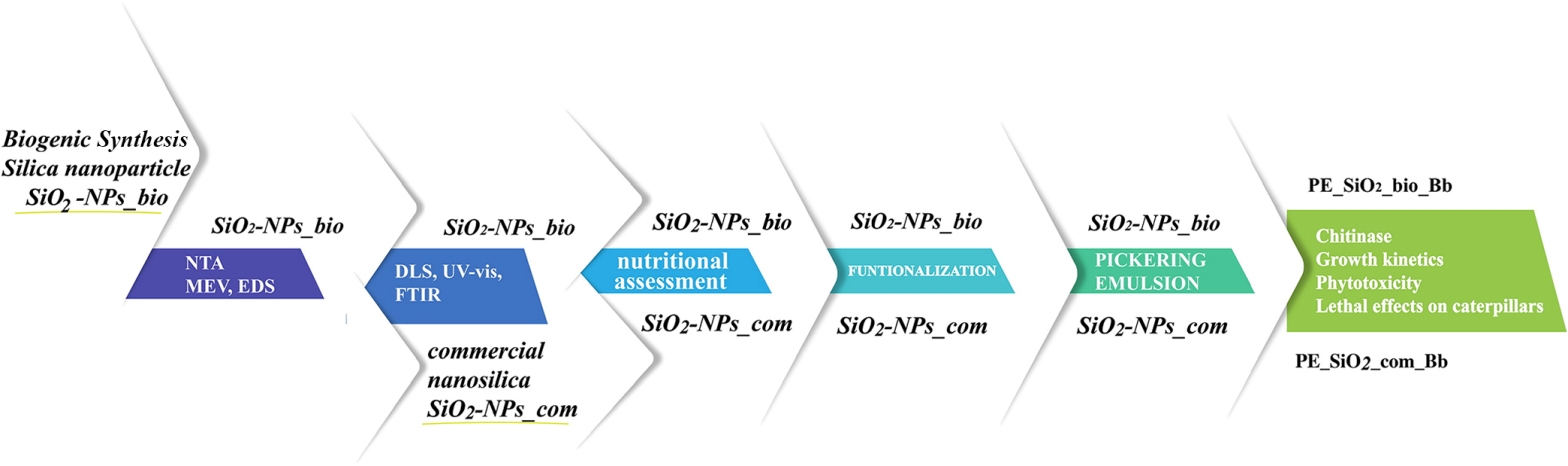
Schematic representation of the activities performed in the study. The biogenic and commercial nanoparticles after formulation and addition of the microorganisms are respectively denominated PE_SiO_2__bio_Bb and PE_SiO_2__com_Bb.

### Characterization of the Nanoparticles

A test for verification of the remaining microorganisms was performed after the synthesis of SiO_2_–NPs_bio. The obtained suspensions were inoculated in potato dextrose agar, and no growth of *F. oxysporum* was observed even after 16 days (Figure S1).

SiO_2_–NPs_bio showed a higher diameter and a lower ζ-potential in comparison with SiO_2_–NPs_com (Table 1). These differences may be attributed to the biogenic synthesis process. In this process, an association of SiO_2_–NPs_bio with the proteins released by the fungus occurs. These proteins interact with the rice husk surface, resulting in morphological modifications.^13,19^ *Fusarium oxysporum* fungus promotes the biolixiviation and biotransformation of silicon into crystalline nanoparticles.^13^ New syntheses were performed to verify the reproducibility of the method, which demonstrated the desired reproducibility.

**Table 1:** Physicochemical Characterization of the Suspensions of Biogenic Silicon Nanoparticles (SiO_2_–NPs_bio) and Commercial Silicon Nanoparticles (SiO_2_–NPs_com) by DLS (Dynamic Light Scattering), Microelectrophoresis, NTA (Nanoparticle Tracking Analysis), and Silicon Content by Reaction with Ammonium Molybdate

	DLS		microelectrophoresis	NTA	silicon concentration	absorbance
suspensions	diameter (nm)	PDI	ζ-potential (mV)	concentration (NPs mL^–1^)	concentration (g/L)	wavelength (nm)
SiO_2_–NPs_bio	324 ± 3	0.3 ± 0.02	–23 ± 0.2	6.12 × 10^10^	64	235
						265
SiO_2_–NPs_com	27 ± 0.5	0.3 ± 0.02	–37 ± 8	4.10 × 10^11^	500	235

Two absorbance peaks at around 235 and 265 nm were observed by UV–vis spectroscopy (Figure S2). Buazar evaluated green silicon nanoparticles synthesized from wheat husk ash and obtained an absorption peak of 235 nm during the formation of the nanoparticles.^20^ Durán et al. attributed the absorption peak around 265 nm to the presence of aromatic amino acids from protein released by *F. oxysporum* during the synthesis.^21^ Then, it is possible to suggest the presence of proteins from the microorganism in the formation of a capping adsorbed to SiO_2_–NPs_bio.

Scanning electron microscopy (SEM) and energy-dispersive X-ray spectroscopy (EDS) revealed dimensions ranging from 60 to 140 nm, with an irregular morphology that was close to spherical or elongated spherical for SiO_2_–NPs_bio (Figure 2).

**Figure 2:**
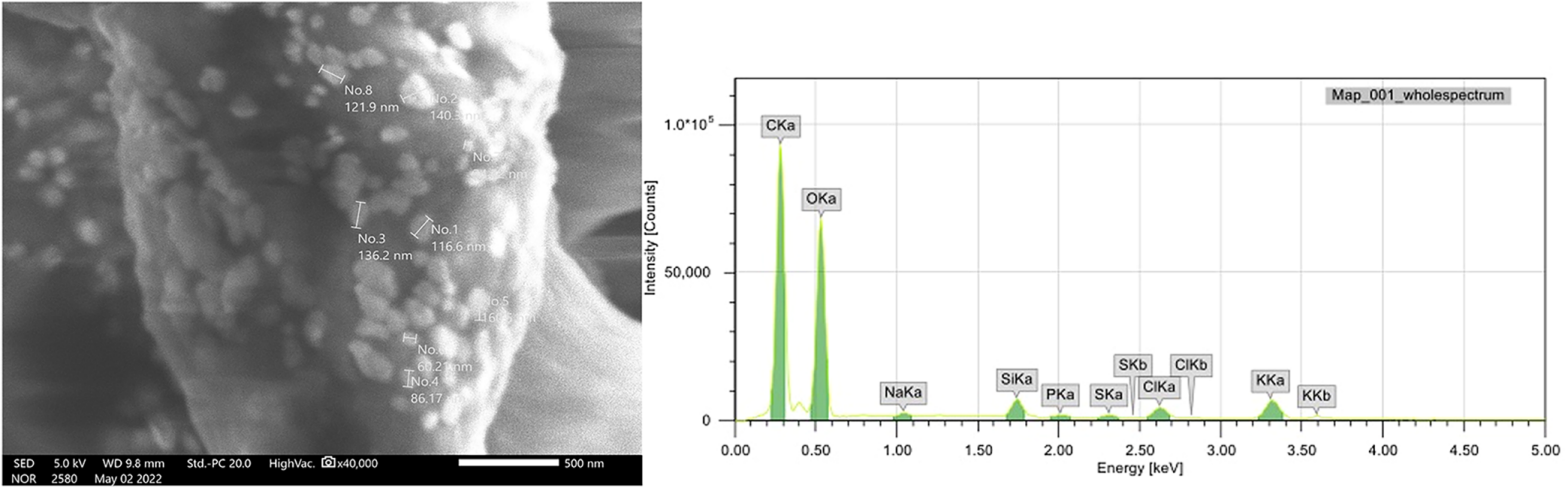
Scanning electron microscopy (× 40.000) of biogenic silicon nanoparticles and its respective EDS peaks. The *Y* axis represents the number of characteristic X-rays that hit the detector, and the *X* axis represents the X-ray energy.

The elemental composition was determined by EDS, and the formation of peaks with different intensities was obtained for carbon (C), oxygen (O), potassium (K), chlorine (Cl), and silicon (Si), which were the main identified elements (Figure 2).

### Investigation of the Functionalization of the Nanoparticles and the Chemical Composition of the Capping by Fourier Transform Infrared Spectroscopy

Fourier transform infrared spectroscopy analysis showed the presence of characteristic functional groups of biomolecules, which were also observed in the filtrate of the nanoparticles (Figure 3).

**Figure 3:**
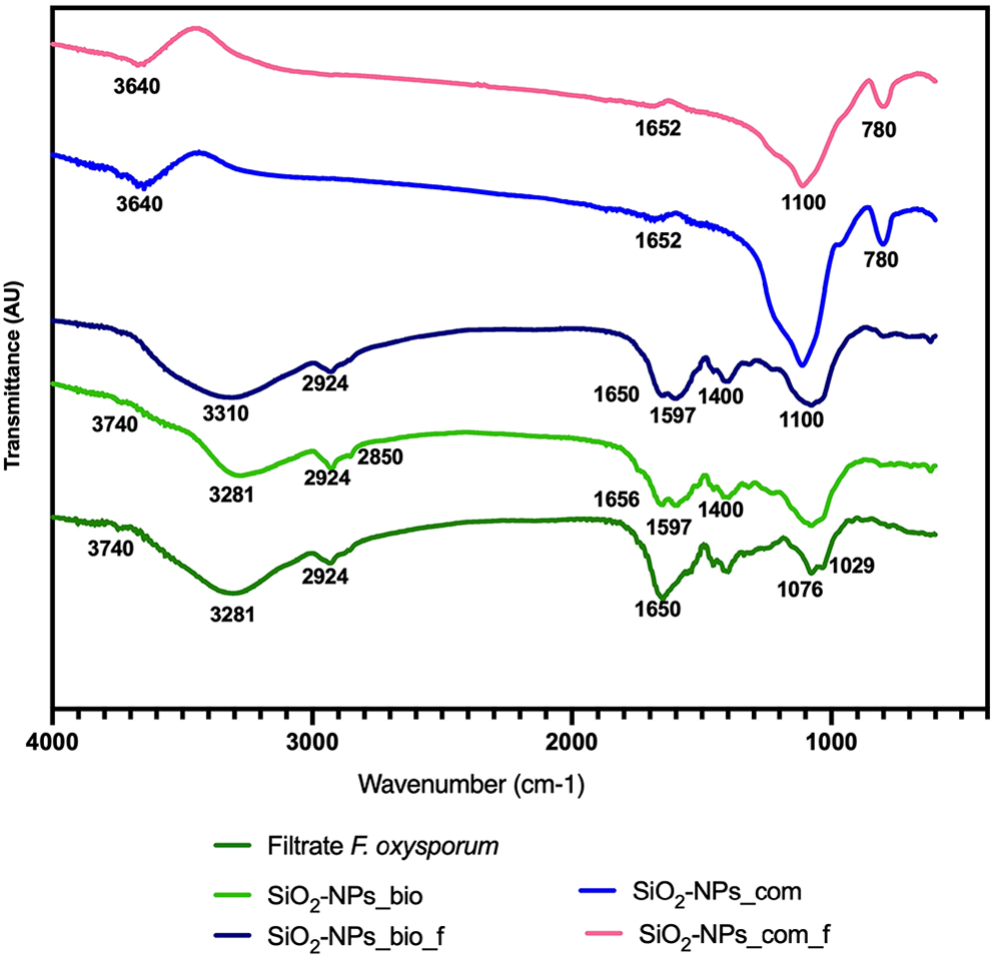
FTIR spectra of the filtrate of *F. oxysporum*; silicon biogenic nanoparticles (SiO_2_–NPs_bio); Functionalized biogenic silicon nanoparticles (SiO_2_–NPs_bio_f); commercial silicon nanoparticles (SiO_2_–NPs_com); and commercial functionalized silicon nanoparticles (SiO_2_–NPs_com_f).

Spectral regions around 460, 810, 960, 1.080–1.200 cm^–1^, and 3.100–3.700 cm^–1^ are associated with the fundamental vibrations of silicon. Bands of 3.550–3.100 cm^–1^ may be related to the hydroxyl group of water (H–O–H) and the silanol group (Si–OH).^22^ It is possible to observe a difference in the intensity of the spectral region which refers to the hydroxyl group (3550–3100 cm^–1^) in SiO_2_–NPs_com (light blue) and functionalized SiO_2_–NPs_bio (pink).

SiO_2_–NPs_bio (light green) and functionalized SiO_2_–NPs_bio samples (dark blue) showed differences in the intensity of the peaks. The increase in the intensity of the spectral region around 3300–3290 cm^–1^ in functionalized biogenic nanoparticles (dark blue) may be related to the asymmetric and symmetric stretching of amine groups.^23^ In addition, a decrease in the CH_3_ band at approximately 2854 cm^–1^ can be identified for the functionalized SiO_2_–NPs_bio sample (dark blue) in comparison with the nonfunctionalized sample (light green). This behavior suggests the effectiveness of the APTES functionalization process, as the hydrolysis and subsequent polymerization steps involved in this treatment result in a reduced band intensity. Bands related to silanol groups exhibit lower intensity after the functionalization process, indicating the adsorption of organic groups on the silicon surface, which replaces the hydroxyl groups.^22^

*F. oxysporum* filtrate was analyzed (Figure 3, dark green). It was possible to observe that the main peaks of the filtrate may remain (dark blue) or not (light green) in the functionalized biogenic nanoparticles; however, with different intensities, suggesting the presence of structures involved in the biogenic synthesis that form the capping of these nanoparticles. Similar results were obtained by Guilger-Casagrande et al. with the presence of similar functional groups in the filtrate and the nanoparticle sample, indicating the formation of a capping by these groups.^24^

### Evaluation of the Cytotoxicity of the Nanoparticles

Cell viability showed a relative decrease at the end of 48 h (following 24 h of exposure) when cells were exposed to the highest concentrations of the sample (Figure 4). It is likely due to cell rounding and detachment from the surface; however, neither cell line exhibited cell death when exposed to biogenic SiO_2_–NP. Although a pronounced decrease was observed in the exposure of the 3T3 cell line, it may be attributed to the cell line’s sensitivity.

**Figure 4:**
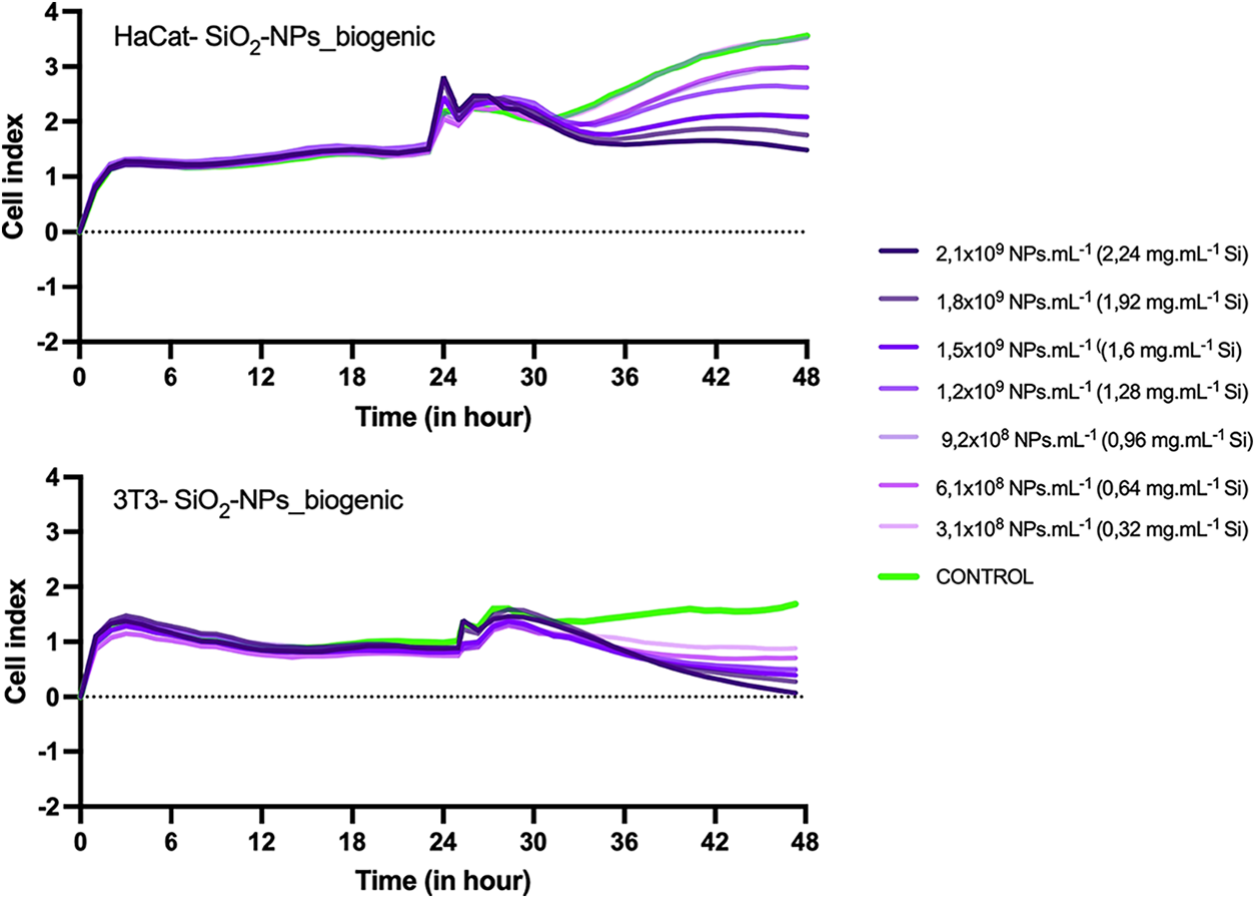
Evaluation of the cytotoxic effects of the suspension of silicon biogenic nanoparticles on HaCat and 3T3 cell lines by real-time cell monitoring assay (RTCA), exposed to different nanoparticle concentrations (NPs mL^–1^) and silicon concentrations (mg mL^–1^).

Cell alterations in HaCaT and V79 cell lines triggered by interactions with metallic biogenic nanoparticles synthesized from the fungus *B. bassiana* using RTCA were not observed in previous studies performed by our group.^24^

### Analysis of Silicon Content and the Effects of Biogenic Silicon Nanoparticles on Plant Photosynthetic Pigments and Nutrients

The absorption of Si by the plants (Figure 5) indicated that the roots showed a lower accumulation than the shoots in the lowest concentration of the element. The concentration of 2 mmol L^–1^ of SiO_2_–NPs_bio showed the highest accumulation of Si, mainly in the shoot, and it was possible to observe that the absorption was inversely proportional to the increase of exposure concentration. This behavior may be due to the higher degree of polymerization of the element, which decreased the absorption of silicate. More studies are necessary to enhance the stability of elements. However, regarding the exposure to SiO_2_–NPs_com, it was possible to observe a high accumulation of Si in the shoots and especially in the roots of the plants exposed to the highest concentrations, with an excessive increase in the concentration of 8 mmol L^–1^.

**Figure 5:**
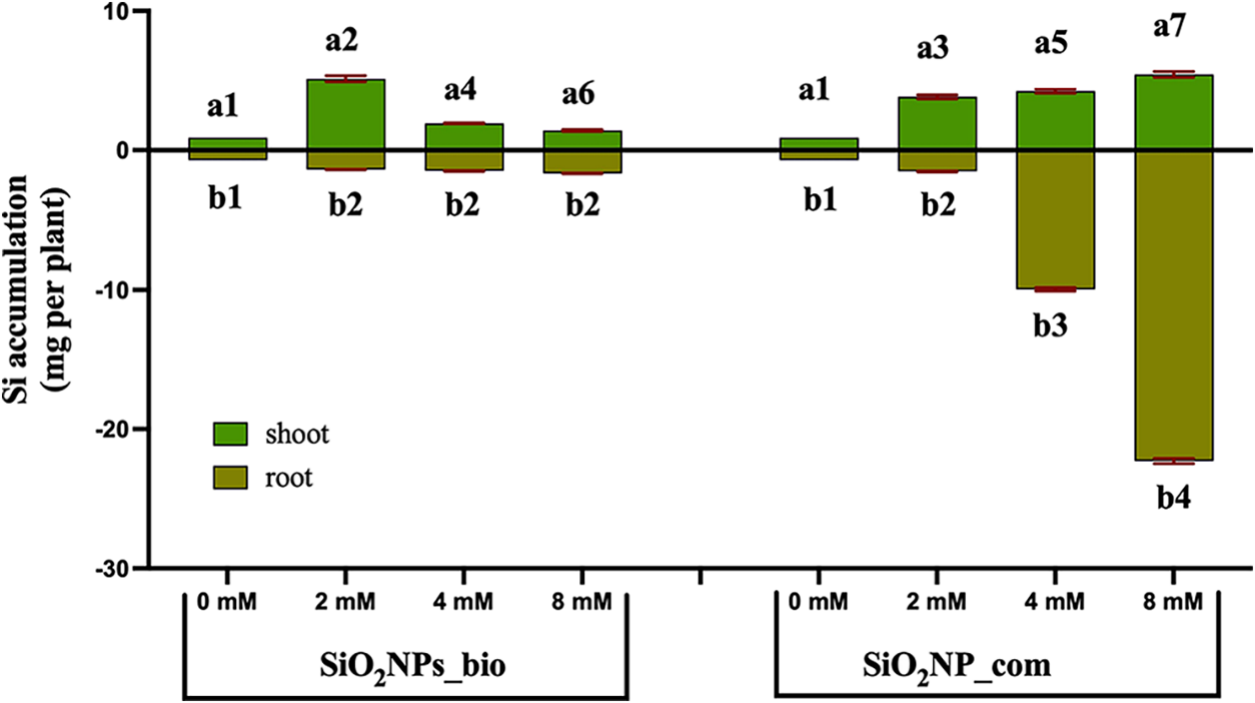
Evaluation of the accumulation of silicon (mg/plant) in the shoot and root of *Solanum aethiopicum*. Statistical analysis: a: shoot, b: root. Different numbers represent a statistically significant difference by Tukey’s test at 5% probability (*p* ≤ 0.05) (*n* = 6).

Although the evaluation was performed only in leaves and roots, it is important to note that in silicon-accumulating plants, the root transporters Lsi1 (influx) and Lsi2 (efflux) mediate the uptake of monosilicic acid and its loading into the xylem for translocation to the shoots. At the nodes, additional transporters such as Lsi6 facilitate intervascular transfer, enabling silicon distribution to different tissues including the seed.^5,25^

It is known that silicon can increase water and nutrient absorption by the roots of some plant species, while maintaining this balance under conditions of drought stress.^26^ Silicon absorption may confer a higher mechanical resistance due to the reinforcement of the cell wall, contributing to the decrease of plant stress.

The exposure of the plants to different concentrations of SiO_2_–NPs_bio and SiO_2_–NPs_com resulted in significant changes in the levels of ascorbic acid, photosynthetic pigments, pheophytin b, total phenolics, and carotenoids (Figure 6) in comparison with the control. Generally, the presence of silicon nanoparticles is associated with an increase of photosynthetic pigments in several plant species.^27^ A summary of the physiological and nutritional effects associated with nanosilica exposure is presented in Table 2.

**Figure 6:**
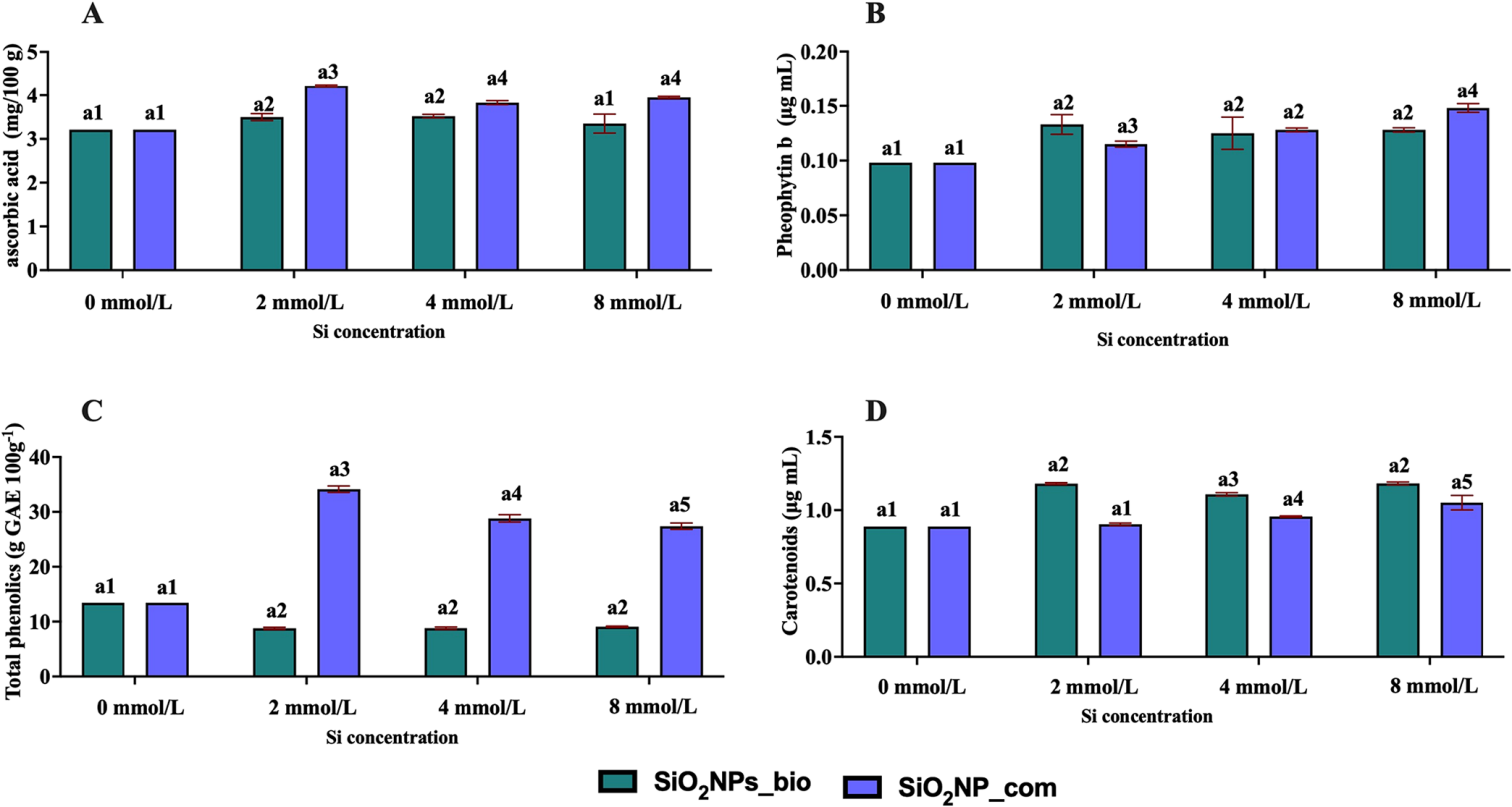
Quantification of (A) ascorbic acid, and photosynthetic pigments (B) pheophytin b, (C) total phenolics, and (D) carotenoids. Statistical analysis: Different numbers represent significant statistical difference by Tukey’s test at 5% probability (*p* ≤ 0.05) (*n* = 6).

**Table 2:** Representative Studies Investigating the Effects of Silicon Nanoparticles on Photosynthetic Pigments and Mineral Nutrient Uptake in Plants

plant species	Si NPs characteristics	application method	experimental contest	effects on photosynthetic pigments	effects on nutrient uptake	major interpretation	refs
*Triticum aestivum* (wheat)	mesoporous silica nanoparticles (MSNs) 20 nm/–22.5 mV	root uptake	seedling growth	↑ total chlorophyll; ↑ photosynthetic activity	not reported	localization in chloroplasts suggests direct enhancement of photosynthetic machinery	^54^
Megathyrsus maximus	silicon nanoparticles (≈10–20 nm; synthetic)	nutrient solution; multiple Si concentrations	phosphorus toxicity	↑ chlorophyll a and b; improved photosynthetic performance	reduced P accumulation; improved nutrient balance	Si NPs mitigated P toxicity by regulating nutrient homeostasis and protecting the photosynthetic apparatus	^55^
*Triticum aestivum*	silicon nanoparticles incorporated into biochar	soil amendment	salinity stress	↑ chlorophyll a and b; ↑ carotenoids; improved gas exchange	↑ N and K; ↓ Na	enhanced salt tolerance via ionic regulation and maintenance of photosynthetic machinery	^41^
*Oryza sativa* (rice)	silica nanoparticles (nanoscale; foliar-compatible formulation)	foliar spray at multiple concentrations	controlled growth conditions	significant increases in chlorophyll content and photosynthetic rate	↑ N, P, and K concentrations in plant tissues	Si NPs promoted coordinated improvement in photosynthesis and mineral nutrition, suggesting enhanced metabolic activity	^43^

Ascorbic acid is considered an antioxidant compound, and in this context, the exposure of plants to nanoparticles caused an increase in the production of this acid, triggering a higher detoxification capacity of reactive oxygen species (ROS) and enhanced photosynthetic activity (Figure 6A). In a study performed by Riaz et al., wheat plants under copper stress were treated with silicon nanoparticles; they demonstrated an increase in the levels of ascorbic acid and other antioxidant compounds, which reduced oxidative damage and enhanced plant growth.^28^ Other studies showed that the exposure of plants under drought stress to silicon nanoparticles increased the concentration of antioxidants, including ascorbate, and plants under saline stress showed an increase in the level of ascorbic acid, suggesting that these nanoparticles may improve the antioxidant potential of the plants and their resilience to environmental stress.^29,30^ It probably occurs by the physiologic and biochemical modulation of the plants and the consequent increase in the antioxidant activity.^30,28,29^

The increase of pheophytin b in plants exposed to SiO_2_–NPs_com (Figure 6C) indicates a higher chlorophyll breakdown as a consequence of a possible foliar senescence.^31^ Some studies report that the presence of silicon nanoparticles may change physiological characteristics of the plants such as the increase of chlorophyll and carotenoid content, suggesting a higher photosynthetic rate.^32,33^ Pheophytin b plays an important role in plant photosynthetic processes, especially in the photosystem II (PSII), since it acts as a primary electron acceptor, contributing to the initial electron transference from chlorophyll to quinone in the conversion of light into chemical energy.^34,35^ Although studies do not directly report the behavior of pheophytin b when exposed to silicon nanoparticles, according to Zahra et al., pheophytin b shows alterations in the case of intoxication by iron.^36^

The content of total phenolics increased when the plants were exposed to SiO_2_–NPs_com, indicating an increase in the plant resistance. In contrast, the plants exposed to SiO_2_–NPs_bio (Figure 6C) showed a decrease in these compounds, indicating a possible vulnerability to stress. In relation to carotenoids, it was possible to observe that the exposure to nanoparticles, especially SiO_2_–NPs_bio, increased the production of this pigment (Figure 6D), indicating photoprotection and the possible enhancement of the mechanisms of dissipation of nonphotosynthetic energy, since studies report that carotenoids are involved in defense against oxidative stress.

A study conducted by Parveen and Siddiqui found that plants exhibited an increase in carotenoid content when exposed to silicon nanoparticles.^37^ It was interpreted as a crucial activity to mitigate the effects of ROS, which are generated under stress conditions and may contribute to better plant development and the decrease of disease levels. A study performed with wheat exposed to low concentrations of silicon nanoparticles increased the production of carotenoids, resulting in a higher photosynthetic efficiency and stress tolerance.^38^

The role of silicon in increasing the synthesis of phenolic compounds^39–41^ can be attributed to the activation of the phenylpropanoid biosynthesis pathway and consequently the production of phenols^42^ from the increase in the activity of the enzyme phenylalanine ammonia-lyase (PAL),^43^ influencing gene expression in the phenylpropanoid pathway.^44^ The relevance of silicon in the synthesis of ascorbic acid^45^ is due to its action on the activity of the key enzyme L-galacton-1,4-lactone dehydrogenase (GLDH).^46^

The increase in these antioxidant compounds induced by silicon has consequences for the preservation of the photosynthetic pigments. The role of Si in increasing carotenoids^47–49^ and especially chlorophyll^50,41^ is due to the fact that Si modulates the expression of genes such as Lhcb3 linked to the synthesis of chlorophyll ab binding protein 387 and also the greater homeostasis of nutrients that make up chlorophyll such as N, Mg, and others.^51^

The increase in chlorophylls and carotenoids increases the capture of light energy.^52^ In the case of an increase in pheophytins, it favors the absorption of wavelengths of light that are not well-captured by chlorophyll, complementing the efficiency of photosynthesis.^53^

Collectively, prior research indicates that silicon nanoparticles can promote photosynthetic efficiency, often coupled to an enhanced nutrient balance and stress alleviation. Nevertheless, these investigations have largely focused on synthetically produced nanoparticles, leaving the physiological implications of biogenic silicon nanomaterials comparatively underexplored.

### Results of Carbon Concentration in Response to Exposure

In relation to carbon concentration, it was possible to observe an accumulation of biomass (Figure 7A), indicating that the increase in nanoparticle concentration triggered a significant increase in the carbon concentration in the exposed plants, as a consequence of higher CO_2_ fixation.

**Figure 7:**
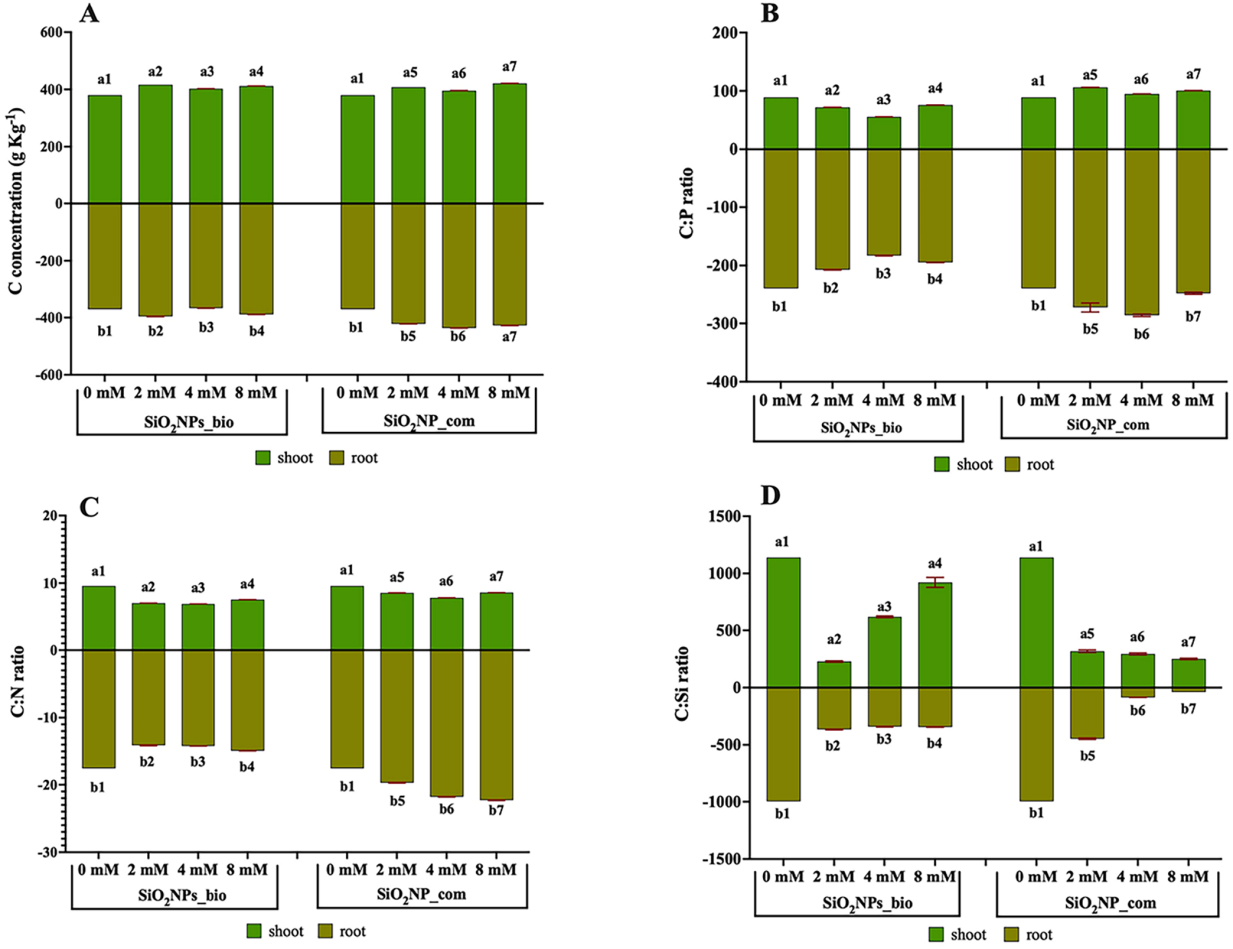
Evaluation of A: carbon concentration (g/kg) and stoichiometric ratio between B: carbon and phosphorus (C:P), C: carbon and nitrogen (C:N), and D: carbon and silicon (C:Si) in *Solanum aethiopicum*. Statistical analysis: a: shoot, b: root. Different numbers represent significant statistical difference by Tukey’s test at 5% probability (*p* < 0.05) (*n* = 6).

The balance between carbon and nitrogen indicates appropriate plant metabolism, and the use of SiO_2_–NPs_bio decreased the C:P ratio. Studies showed that plant exposure to nanoparticles commonly induces an increase in water absorption and changes protein amount, particularly those involved in the routes related to oxidative stress, ROS detoxification, and hormonal regulation, which may alter the balance of carbon and nitrogen metabolism.^56^

The stoichiometric C:P ratio indicates that SiO_2_–NPs_bio improved the energetic status of the plants with a higher synthesis of phospholipids. It was not observed in plants exposed to SiO_2_–NPs_com, whereas an increase in the C:P ratio was observed for all of the tested concentrations, which indicates a possible energetic limitation (Figure 7B).

The use of Si nanoparticles increases the absorption of nitrogen and phosphorus and, consequently, modulates the C:N ratio, increasing nitrogen content. It is related to the higher photosynthetic efficiency and biomass production since silicon enables a better carbon use efficiency and nutrient cycling.^57^ Other studies reported that silicon nanoparticles promote the expression of organic compounds such as proteins and chlorophyll, which are essential for photosynthesis and plant growth.^58^ Higher nitrogen absorption and protein synthesis indicate better physiological conditions that trigger a better resistance of the plants against environmental stress.^59,60^

In addition, the use of silicon nanoparticles increases the activity of defense enzymes,^59^ supporting plant resilience to biotic and abiotic stress, improving water absorption, and contributing to plant growth.^60^ These beneficial changes highlight the potential of silicon nanoparticles for agricultural applications.^27,55,47,48^

It is possible to conclude that SiO_2_NPs_bi was absorbed by the plant and improved some physiological attributes (Figure 8). Although the plants exposed to SiO_2_NPs_com showed better results, SiO_2_NPs_bio may be a sustainable alternative for a higher plant yield. The results indicated that the plants exposed to SiO_2_NPs_com showed a higher dry weight than those exposed to SiO_2_NPs_bio (Figure 8A). Studies performed by Alam et al. reported that tomato plants under saline stress showed better growth and better physiological parameters, such as photosynthesis and antioxidant activity, leading to an increase in dry weight when exposed to silicon nanoparticles.^61^

**Figure 8:**
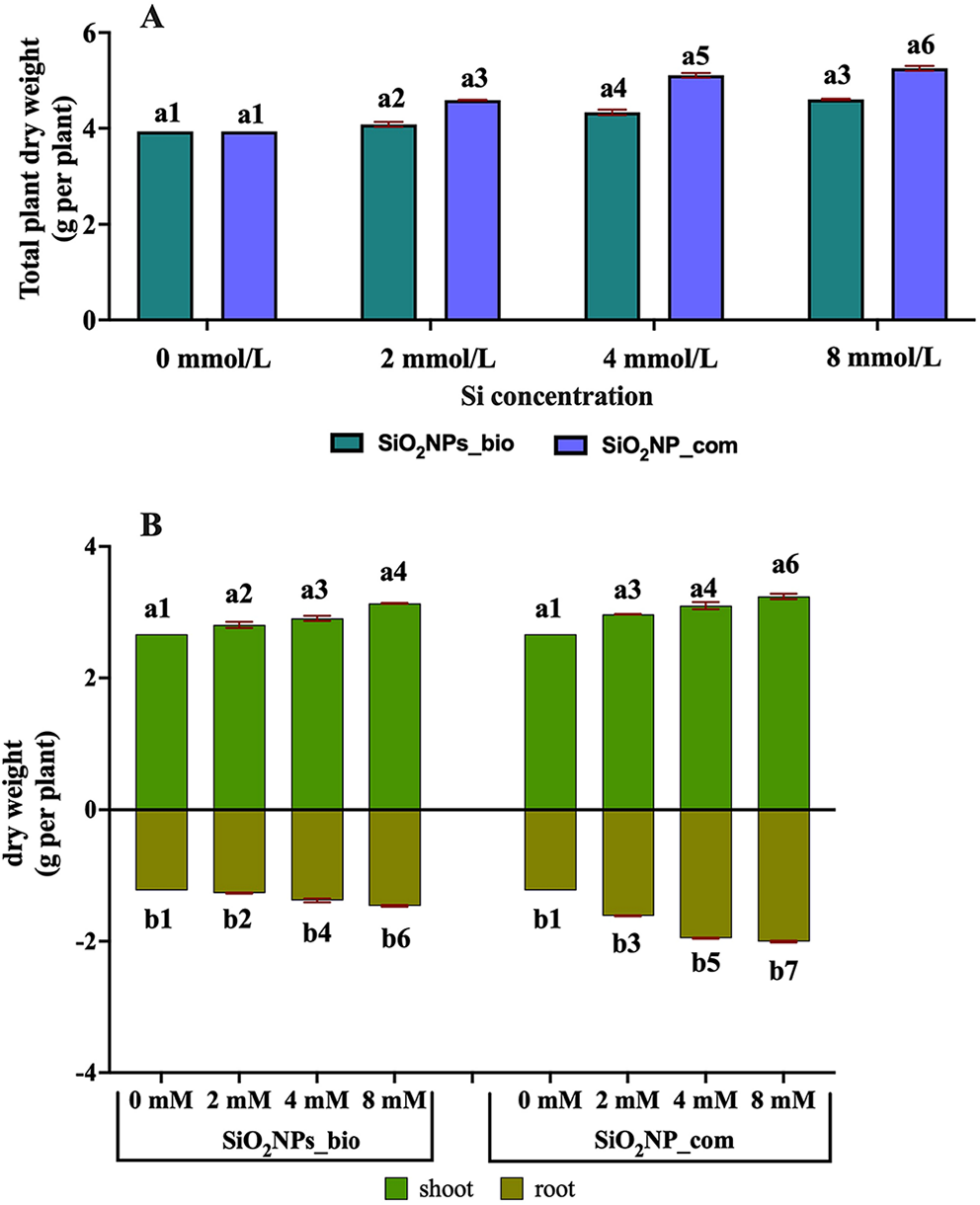
Evaluation of the biomass of *Solanum aethiopicum*, A: Total plant dry weight and B: Shoot and root dry weight. Statistical analysis: a: shoot, b: root. Different numbers represent a significant statistical difference by Tukey’s test at 5% probability (*p* < 0.05) (*n* = 6).

Another study reported an increase of the total dry weight of peanut plants exposed to nanosilicon by foliar spray application, occasioned by a better photosynthetic efficiency.^62^ An increase of dry weight was also observed in corn plants exposed to nanosilicon, which is attributed to the higher absorption of nutrients under sodium-saline conditions of the soil.^63^ Mesoporous silicon nanoparticles (MSNs) increased the biomass of *Arabidopsis thaliana* plantlets under nonstress conditions, demonstrating its potential to enhance plant growth even in the absence of stress.^64^

Then, in general, the role of silicon nanoparticles is highlighted in the promotion of vegetative growth and increase of biomass of several plant species, in addition to the increase of photosynthetic mechanisms and stress tolerance.^65,59^ Thus, the application of Si NPs seems to be a promising strategy to increase plant dry weight, contributing to a better crop yield and agricultural sustainability.

### Characterization of the Pickering Emulsion

After a screening of formulation preparation, the best products were those composed by 6% of functionalized SiO_2_-NPs and a 3:7 oil–water proportion (O/A), which were denominated PE-SiObi376 and PE-SiOcom376 (Figure S3).

The combination of SiO_2_-NPs (Pickering emulsion) and *B. bassiana* (10^8^ conidia mL^–1^) was performed for the encapsulation, and the product was denominated PE_SiO_2__bio_Bb (Figure 3s) and PE_SiO_2__com_Bb, according to the used nanoparticles (Table 3).

**Table 3:** Correspondence of Nanoparticles, Emulsion Preparation, and Addition of the Fungus

silica nanoparticles	Pickering emulsion nanoformulation	emulsion + *B. bassiana* consortia
SiO_2_-NPs_bio	PE-SiObio376	PE_SiO_2__bio_Bb
SiO_2_-NPs_com (LUDOX)	PE-SiOcom376	PE_SiO_2__com_Bb

### Chitinolytic Activity of *B. bassiana* Formulations

The activity of the Chitinase enzyme was evaluated by a plate assay (Figure 9). The importance of Chitinase is linked to the infection of hosts, as it facilitates the penetration of the fungus through the degradation of chitin, a polysaccharide that composes the cell wall of microorganisms and the exoskeletons of insects.^66^

**Figure 9:**
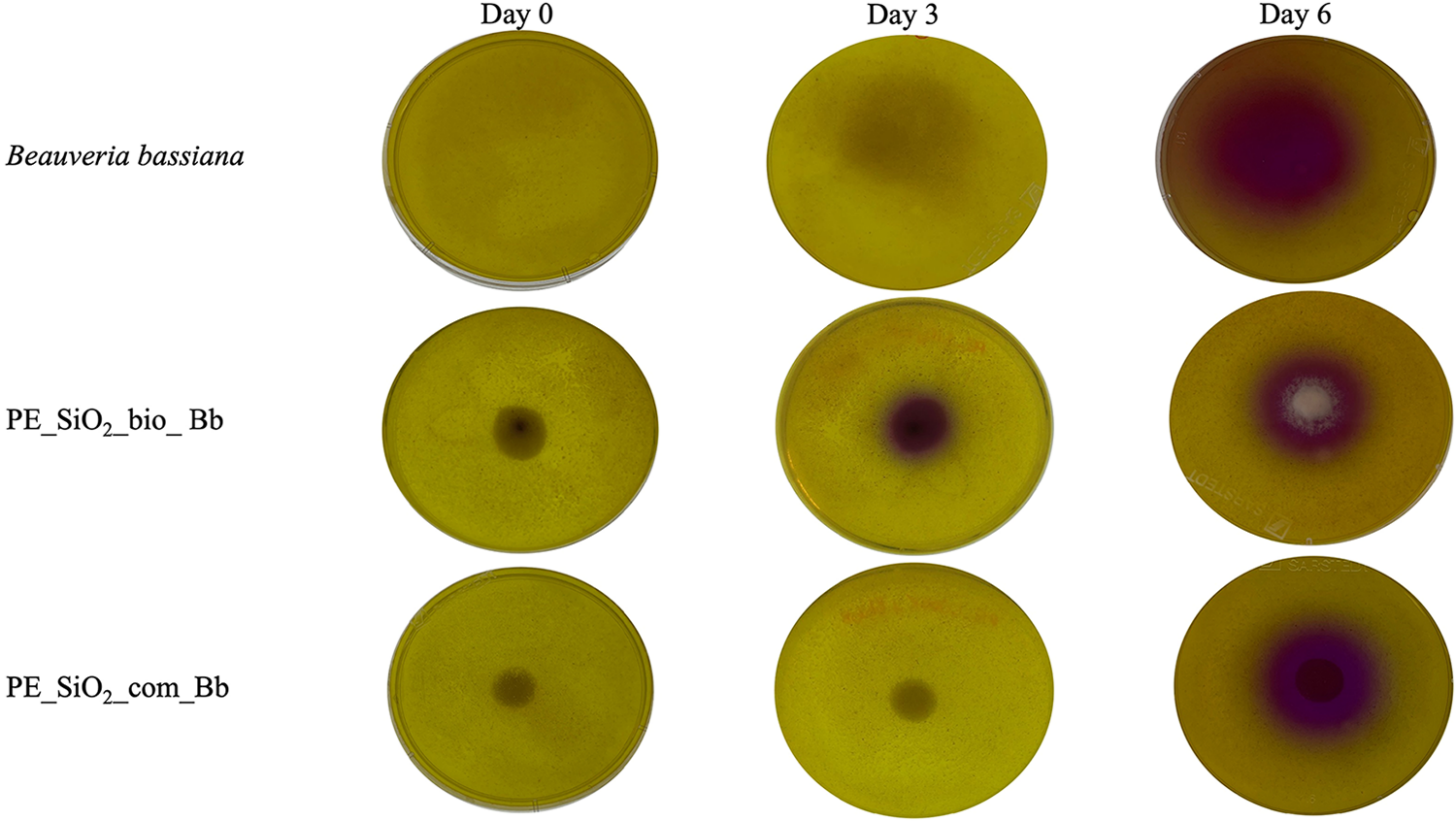
Evaluation of the chitinolytic activity in agar plates. Formulation with biogenic (PE_SiO_2__bio_Bb) and commercial (PE_SiO_2__com_Bb) silicon nanoparticles containing *Beauveria bassiana* fungus and control with the isolated fungus at initial time, 3 days, and 6 days after plating.

The activity of hydrolytic enzymes may be associated with the preserved viability of the fungus after incorporation into the microemulsion, whereas the obtained concentration may be due to the higher fungal growth and the slow release of the encapsulated microorganism. The induction of Chitinase production by silicon was observed by Chérif et al.,^67^ possibly justifying the higher production of the enzyme in the purple plates (Figure 9, day 3) and the higher purple intensity for sample PE_SiO_2__bio_Bb.

Studies showed that *B. bassiana* produces extracellular Chitinase during the logarithmic growth phase. According to Elawati et al., the enzymatic activity reaches the peak of 0.585 U.mL^–1^ on the fourth incubation day, indicating its role in primary metabolism and suggesting its importance in fungal life cycle and pathogenicity.^68^

This production may be enhanced by different strategies such as the optimization of the cultivation conditions such as specific carbon and nitrogen sources, pH, and temperature, which significantly increase the activity of Chitinase and, consequently, fungal efficiency.^69,70^ Then, the exposure of *B. bassiana* to silicon nanoparticles may alter this production of Chitinase.

#### Evaluation of Growth Kinetics of Encapsulated *B. bassiana*

The evaluation of the behavior of nonencapsulated *B. bassiana* (Bb 10^8^) and encapsulated into a combined encapsulation emulsion, with biogenic and nonbiogenic silicon nanoparticles (Figure 10), showed the behavior of the samples in the presence and absence of UV radiation. The results showed clearly the absence of development of *B. bassiana* and death under exposure to UV; this was not observed in the nanoformulations.

**Figure 10:**
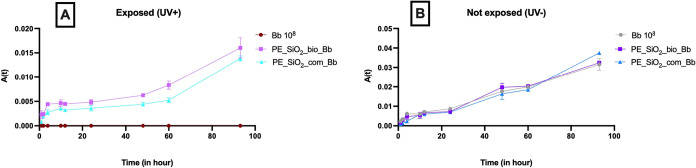
Mycelium growth as a function of time. *B. bassiana* (Bb) suspension 10^8^ conidia. mL^–1^, pickering emulsion containing biogenic silicon nanoparticles and *B. bassiana* 10^8^ (PE_SiO_2__bio_Bb) and pickering emulsion containing commercial silicon nanoparticles and *B. bassiana* 10^8^ (PE_SiO_2__com_Bb), A: exposure to UV light with 12 h photoperiod and B: control, without exposure to UV radiation (*n* = 2).

Solar radiation is a very impactful factor on the viability of biological control fungi after application.^71^ Silicon nanoparticles covering the biological agent promoted protection against UV radiation, triggering a higher bioavailability of the fungus.

Karunakaran et al. reported that nanosilicon improves the content of nutrients in the soil and the conditions for the growth of beneficial bacterial populations, which could indirectly benefit *B. bassiana* with the creation of a more favorable growth environment.^72^ Shatalova et al. showed that silicon nanoparticles combined with *B. bassiana* showed a synergistic effect and significantly reduced pest populations in a more efficient way compared to any isolated treatment.^73^

Generally, the incorporation of silicon nanoparticles and *B. bassiana* improves the efficiency against pests and can also be a sustainable and efficient alternative to chemical products.

### Evaluation of the Phytotoxic Potential of the Microemulsions

The analysis of the pre-emergence treatments (Figure 11) did not show significant differences in comparison with control and the other samples, with the absence of harm for the development of shoot and root. In the post-emergence treatments (Figure 12), it was possible to observe an increase in shoot fresh and dry weight (Figure 12D) of the plants exposed to pickering emulsions, both with the biogenic nanoparticles (PE_SiO_2__bio_Bb) and the commercial silicon nanoparticles (PE_SiO_2__com_Bb). This result may be attributed to the absorption of silicon nanoparticles by the leaves, which can enhance plant growth and optimize the functions of some plant species under stress and in the absence of these conditions, supposedly by the interactions with phytohormones, antioxidants, and other signaling molecules.^74^

**Figure 11:**
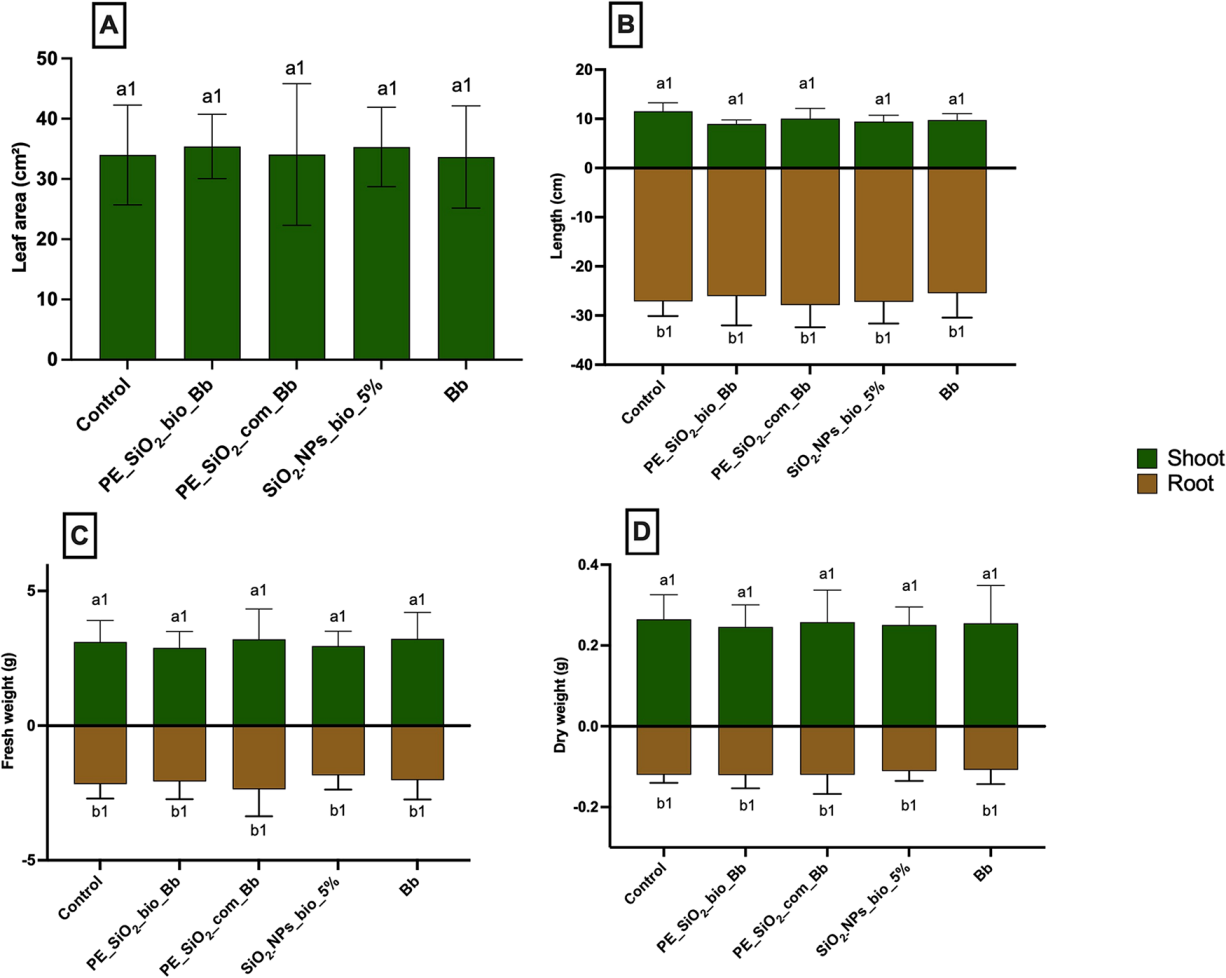
Evaluation of the effects of pre-emergence treatments on the morphological parameters of *Phaseolus vulgaris* plants. A: Shoot and root; B: leaf area; C: shoot and root fresh weight; D: shoot and root dry weight. Statistical analysis: a: shoot, b: root. Different numbers represent significant statistical difference by Tukey’s test at 5% probability (*p* < 0.05), *n* = 5.

**Figure 12:**
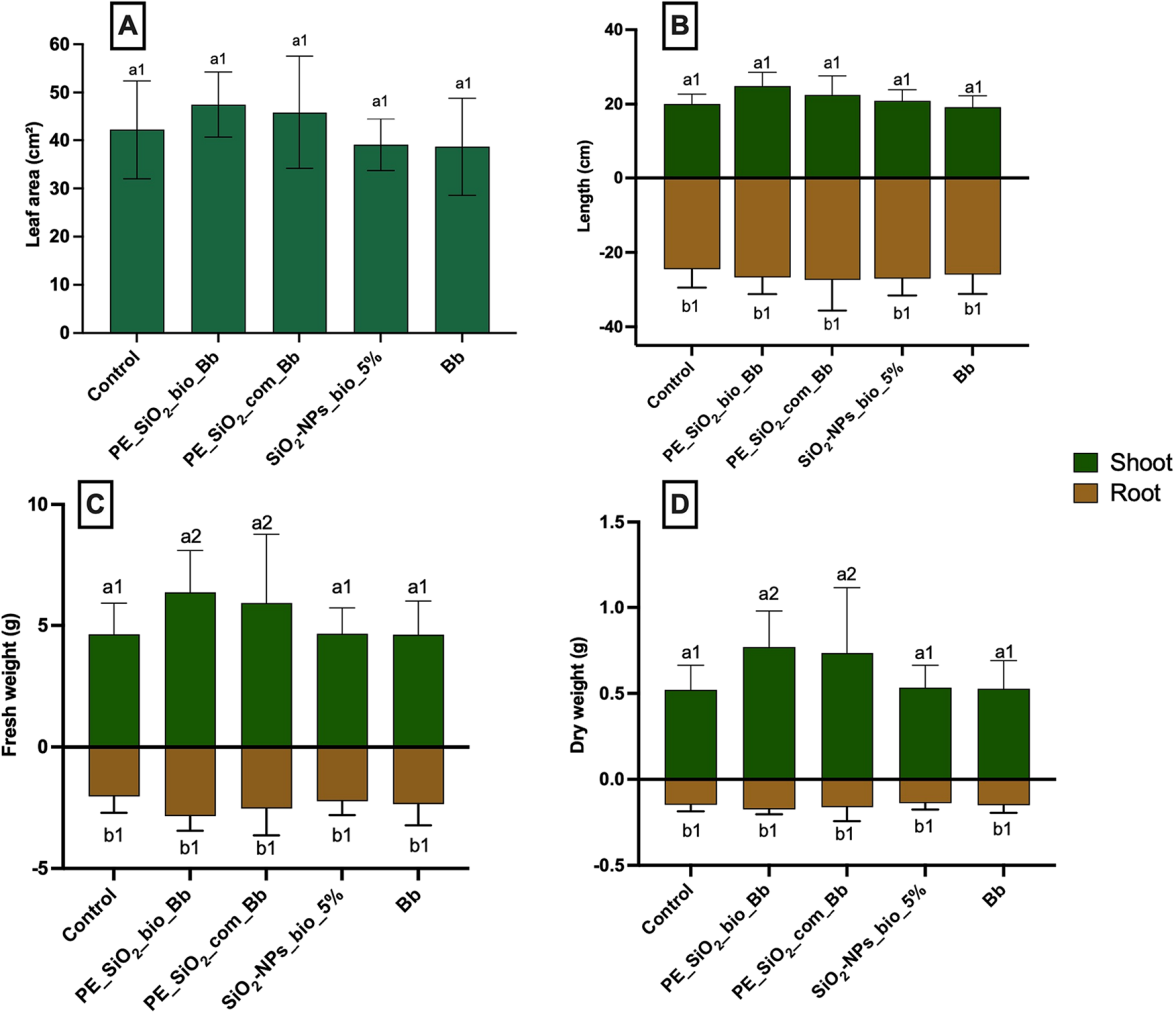
Post-emergence evaluations. A: Shoot and root; B: leaf area; C: shoot and root fresh weight; D: shoot and root dry weight. Statistical analysis: a: shoot, b: root. Different numbers represent significant statistical difference by Tukey’s test at 5% probability (*p* < 0.05), *n* = 5.

In the other analysis, no significant statistical difference was observed; however, it is possible to observe a slight contribution of these samples for the development of the leaf area of the plantlets (Figure 12A) as well as shoot length (Figure 12B).

It is worth mentioning that in this study, plant cultivation was performed in a short time, whereas it is not possible to discard the possibility of better long-term results.

The oxidative stress markers MDA and H_2_O_2_ were analyzed for both pre- and post-emergence treatments (Figure 13).

**Figure 13:**
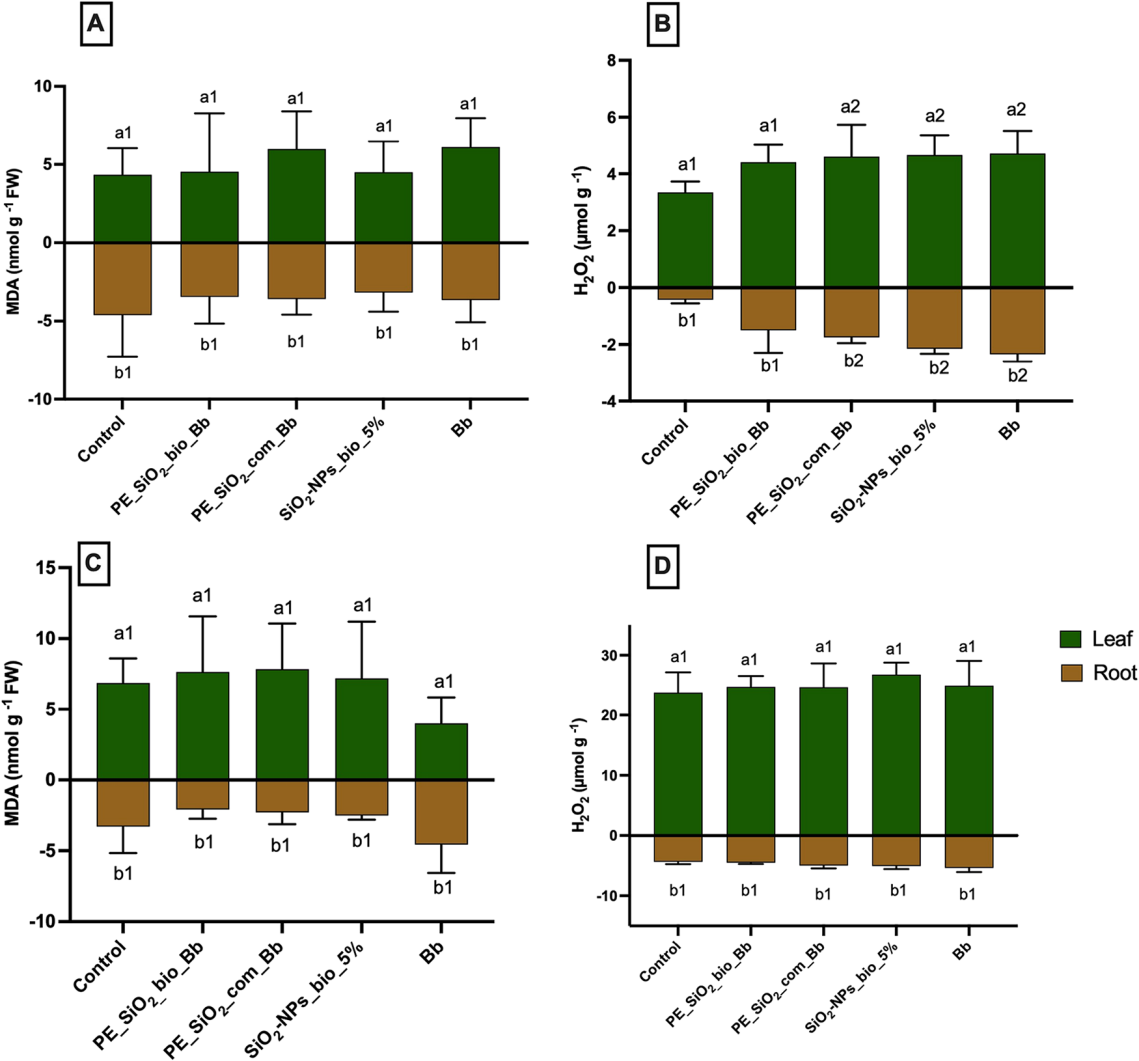
Pre-emergence evaluation of A: Leaf and root MDA; B: leaf and root H_2_O_2_ and post-emergence evaluation of C: leaf and root MDA and D: leaf and root H_2_O_2_. Statistical analysis: a: shoot, b: root. Different numbers represent significant statistical difference by Tukey’s test at 5% probability (*p* < 0.05). *n* = 5.

No significant changes were observed in the level of MDA in the leaves and roots for the pre-emergence treatments in comparison with the control (Figure 13A). Similar results were observed for the post-emergence treatment (Figure 13C), indicating the absence of alterations in the levels of lipid peroxidation. However, increased levels of H_2_O_2_ were observed for the pre-emergence treatment in comparison with the control (Figure 13B). These increases comprehended 37, 39, and 41% in leaves exposed to pickering emulsion with combined encapsulation of commercial nanosilicon and the biological agent (PE_SiO_2__com_Bb), a 5% suspension of silicon biogenic NPs (SiO_2_–NPs___bio_5%), and a suspension containing only the biological agent (*B. bassiana*), respectively. The increase in H_2_O_2_ levels was more pronounced in the roots, indicating the induction of oxidative stress by oxygen reactive species in this organ.

In the pre-emergence treatment, the pickering emulsion composed of the biogenic nanoparticles synthesized in this study (PE_SiO_2__bio_Bb) did not cause significant alterations in shoot and root levels of H_2_O_2_ in comparison with the control (Figure 13B).

### Lethal Effects of the Nanoparticles and Formulations in *Spodoptera frugiperda, Spodoptera cosmioides*, *Chrysodeixis includens*, and *Rachiplusia nu*

The results of the evaluation of the lethal effects of the pickering emulsion with combined encapsulation of biogenic SiO_2_–NPs and *B. bassiana* (10^8^ conidia mL^–1^) (PE_SiO_2__bio_Bb); pickering emulsion with combined encapsulation of commercial SiO_2_–NPs and *B. bassiana* (10^8^ conidia mL^–1^) (PE_SiO_2__com_Bb); biogenic silicon nanoparticles (6.12 × 10^10^ NPs mL^–1^); and a fungal suspension of *B. bassiana* (10^8^ conidia mL^–1^) (Bb) are expressed as a percentage in Figure 14. Dead insects showed no variation in symptoms across the treatments.

**Figure 14:**
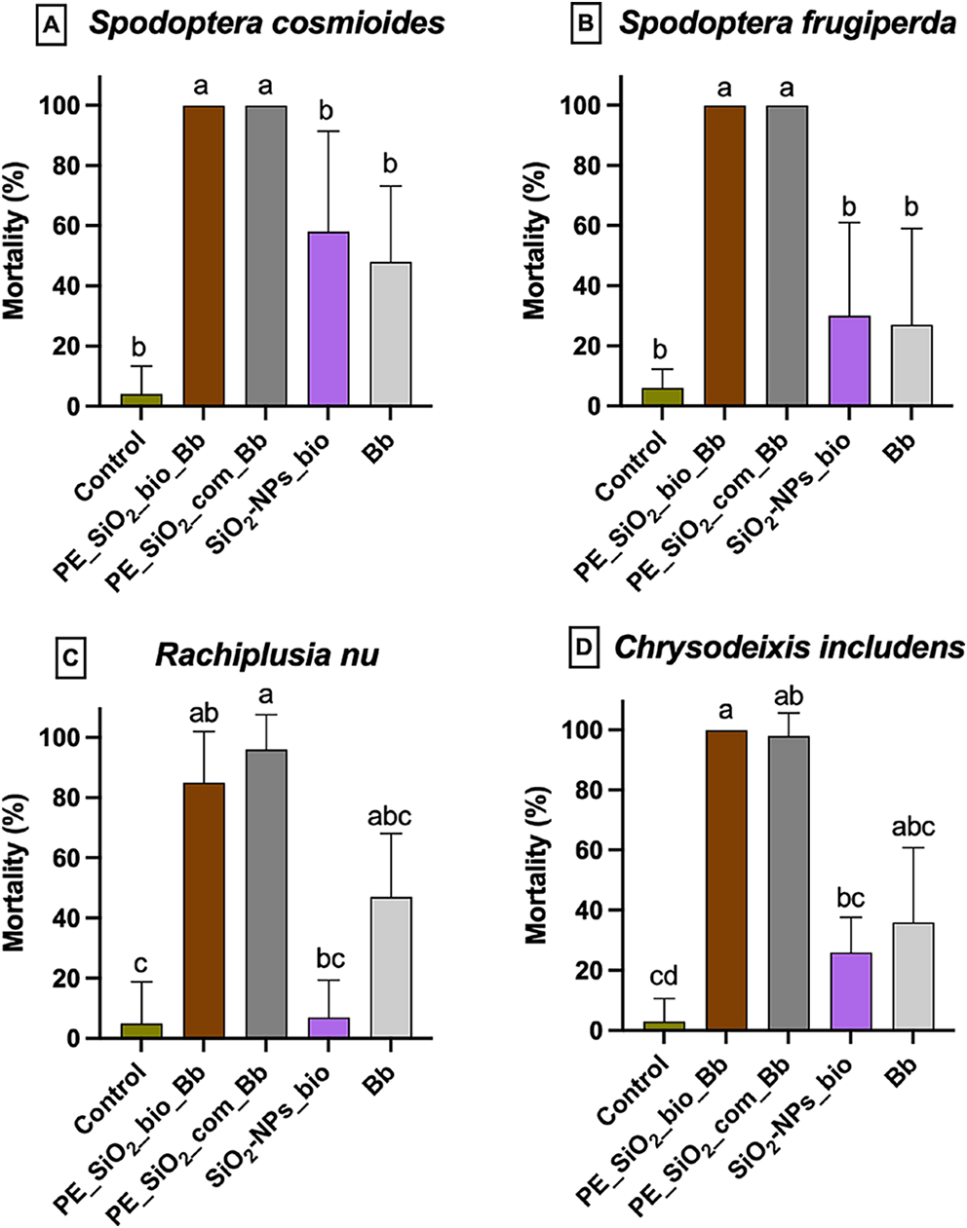
Lethal effects of the formulations containing *B. bassiana*, the suspensions of biogenic silicon nanoparticles and *B. bassiana* in (A) *Spodoptera cosmioides*, (B) *Spodoptera frugiperda*, (C) *Rachiplusia nu*, and (D) *Chrysodeixis includens*. Means followed by different letters are statistically different (Dunn’s test: *p* < 0.05). Bars represent mean ± SEM (*n* = 5).

All species showed high susceptibility to the treatments PE_SiO_2__bio_Bb and PE_SiO_2__com_Bb, with mortalities above 80%. This is very relevant for species such as *S. frugiperda* and *R. nu*. This result supports the findings of this study, suggesting an equivalence between microemulsions containing biogenic nanoparticles and those with nonbiogenic nanoparticles. In addition, a special advantage is conferred on the microemulsion containing biogenic SiO_2_ NPs, as it enables the development of a formulation through an environmentally friendly and low-cost process.

*Spodoptera frugipeda* and *Rachiplusia nu* showed a high susceptibility to the nanoparticles. The first one is a highly polyphagous species that shows frequent resistance to insecticides and Bt plants. Additionally, it has a significant destructive capacity in several crops worldwide, with recent occurrences reported in Asia and Europe.^75,76^ The second species emerged as a problem for soybean culture in Brazil because of recent and unexpected reports of resistance to Bt plants, implying the need for chemical insecticide applications and increasing control costs.^77^

## Conclusion

The present study demonstrates that the biogenic synthesis of nanoparticles using rice husk as a precursor of silicon through interaction with the inactive biomass of the fungus *F. oxysporum* is a viable process as well as its application in more elaborate formulations. Nanosilicon formulation triggered the increase of the viability of *B. bassiana*, validating the development of a potential nontoxic and beneficial vehicle for the optimization of the application of this microorganism as a biopesticide, with no crop damage. These promising results open up perspectives for future studies aiming to optimize the production of nanoparticles in a solid state as well as the exploitation of the potential applications of silicon nanoparticle suspensions in diverse processes, especially as a nanofertilizer, since it possesses a considerable silicon content and low toxicity.

## Methods

### Biogenic Synthesis of Silicon Nanoparticles

Based on the adapted methodology of Bansal et al.,^13^ the initial cultivation of *Fusarium oxysporum* was performed on potato dextrose agar for 5–7 days at 28 °C. After fungal growth, the mycelium disks were transferred to potato dextrose broth, and the culture was maintained under orbital stirring at 150 rpm and 28 °C for 7 days. Then, the biomass was collected by filtration, washed, and added into a mixture with the proportions of 20 g of fungal biomass, 10 g of rice husk, and 100 mL of ultrapure water. This mixture was maintained under the previous conditions, with increased stirring at 200 rpm, for 24 h. Then, vacuum filtration was performed to separate the biomass and rice husk from the aqueous solution. The resulting filtrate was treated with a phenol-chloroform solution (1:1; v/v) and centrifuged at 9000 rpm for 10 min. The supernatant containing the nanoparticles was collected.

#### Functionalization of the Nanoparticles

The suspensions of SiO_2_–NPs_bio and SiO_2_–NPs_com were previously lyophilized for functionalization. 40 mL of absolute ethanol was added to the suspensions under stirring at room temperature. Then, the 3-aminopropyltriethoxysilane (APTES) reagent was slowly added to achieve a final concentration of 0.5 M, and the mixture was kept under stirring under the same conditions for 3 h. The nanoparticles were collected by centrifugation at 9000 rpm for 10 min, washed four times with absolute ethanol, and dried at 35 °C in a vacuum for 3 days. APTES is a coupling agent that possesses the capacity of binding to chemically reactive amine groups in silicon substrates, activating its surface.^23^

#### Preparation of the Microemulsion (Pickering Emulsion)

Ultrapure water and mineral oil, in proportions of 3:7, respectively, were used in the composition of the pickering emulsion. For the stabilization of the mixture, the SiO_2_–NPs, previously functionalized with APTES, were tested at concentrations of 3 and 6%. The mixtures were sonicated to promote the emulsification. The emulsions were prepared with commercial and biogenic nanosilicons. The formulations were evaluated for their ability not to present breakage, flocculation, or coalescence. After obtaining the optimized formulation, the suspension of *B. bassiana* in a concentration of 10^8^ conidia mL^–1^ was incorporated into the formulation by vortexing for 5 min.

#### Physicochemical Characterization of the Nanoparticles

Dynamic light scattering (DLS) and microelectrophoresis techniques were employed to determine the hydrodynamic diameter, polydispersity index, and ζ-potential of the nanoparticles. The samples were analyzed using a ZetaSizer Nano ZS 90 (Malvern) at a fixed angle of 90° and 25 °C. The concentration of the samples in nanoparticles per milliliter (NPs/mL) was determined by nanoparticle tracking analysis (NTA) using a NanoSight LM10, equipped with a 532 nm wavelength laser (green), a CMOS camera, and NanoSight software (version 3.1). The scanning of the nanoparticle suspensions was performed using UV–vis spectroscopy (Agilent model Cary 60) between 200 and 400 nm.

The morphology of the nanoparticles was analyzed by scanning electron microscopy (SEM) technique, using an electronic microscope connected to a system of energy-dispersive X-ray spectrometry (EDS) (Jeol, JSM-IT 200A model). The samples were deposited in stubs and coated with colloidal gold through a pulverization technique. The micrographs were obtained by random scanning. The samples were characterized by Fourier transform infrared spectroscopy (FTIR) to verify and identify the present functional groups and primarily to evaluate the characteristics of the silicon nanoparticles (both biogenic and commercial) after functionalization with APTES. The filtrate of *F. oxysporum* employed in the synthesis process was also analyzed. For the analysis, KBr tablets were prepared in the proportion of 98.5 mg of KBr and 1.5 mg of samples. The analysis was performed in the range of 4000–400 cm^–1^, using JASCO-FT/IR-410 equipment.

#### Evaluation of the Cytotoxicity of the Nanoparticles

The analysis of real-time cell monitoring by electrical impedance, RTCA (Real-Time Cell Analyzer), was performed with HaCat (human keratinocytes) and 3T3 (Swiss albino mouse). The assay was conducted by plating the cells in E-plates (Agilent) in the concentrations of 35, 30, 25, 20, 15, 10, and 5% of NP suspension, which corresponds to the concentrations of 2.5 × 10^9^, 1.8 × 10^9^, 1.5 × 10^9^, 1.2 × 10^9^, 9.2 × 10^8^, 6.1 × 10^8^, and 3.1 × 10^8^ NPs mL^–1^, respectively. The cell behavior was monitored by the xCELLigence system at 37 °C in 5% CO_2_ atmosphere, considering the initial 24 h as a period for cell adhesion. Samples were added after cell adhesion, and the exposure was monitored for 24 h, totaling 48 h.

#### Analysis of Silicon Content and the Effects of Biogenic Silicon Nanoparticles on Plant Photosynthetic Pigments and Nutrients

The analysis of silicon content in the biogenic SiO_2_–NPs was performed according to Korndörfer et al. through the colorimetric method using a yellow silicomolybdic complex and UV–visible spectrophotometry (DU640, Beckman-EUA) at a wavelength of 410 nm.^78^

Nutritional and physiological parameters were evaluated by comparing SiO_2_–NPs_bio and SiO_2_–NPs_com. For this purpose, *Solanum aethiopicum* was used as a model plant due to its superior ability to absorb silicon among the species available to conduct the analysis. The adapted methodology of Hoagland & Arnon was followed.^79^ Both the nanoparticles, in the concentrations of 0, 2, 4, and 8 mmol of silicon per liter, were added into the nutrient solution composed by nitrogen (15 mmol L^–1^), phosphorus (1 mmol L^–1^), potassium (6 mmol L^–1^), calcium (4 mmol L^–1^), magnesium (2 mmol L^–1^), sulfur (2 mmol L^–1^), boron (46 μmol L^–1^), iron (90 μmol L^–1^), manganese (12.6 μmol L^–1^), molybdenum (0.1 μmol L^–1^), and zinc (1.3 μmol L^–1^), with pH adjusted to 5.5–6.0. This analysis aimed to evaluate the silicon absorption by the plant and verify the effects on nutritional homeostasis, growth, and plant resistance.

The study was performed in greenhouse (15 m length × 6 m width × 4 m height in the highest point and 3 m height in the lowest point, with a glass roof) in the Department of Soil Science of the Faculty of Agricultural and Veterinary Sciences of the São Paulo State University, in Jaboticabal city (−48.3224 21° 15′ 19″ South, 48° 19′ 21″ west, 615 m altitude), São Paulo, Brazil. After 15 days of acclimatization in the greenhouse, the plantlets with four leaves were transferred to 1.5 L pots containing washed white sand. The sand was submitted to an acid treatment with 0.5 L of 0.1 M HCl solution to eliminate organic contaminants.^40^ The pots were weekly washed with 1.5 ± 0.25 L of deionized water to avoid salt accumulation until the reduction of electric conductivity to less than 0.010 dS m^–1^. Initially, during the first experiment, a daily application of biogenic silicon as a source of Si was performed for 30 days. Then, plants were cleaned, weighed, and separated for the analysis. The same process was adopted for the SiO_2_–NPs_com.

For the analysis of pigments and ascorbic acid, ten leaf discs, measuring 26.4 mm^2^ each, were collected from the middle third of the blade of the second fully developed leaf and immediately weighed to evaluate the fresh mass. The samples were then depigmented with an 80% acetone. The analysis was based on spectrophotometry with specific wavelengths: 470 nm for carotenoids, 665 nm for pheophytin, and 653 nm for pheophytin b.^80^ The total phenolic compound content was determined by using fresh leaf samples (54 days post-transplantation). The phenol content was determined by a colorimetric reaction induced by the Folin-Ciocalteu reagent, following the methodology of Singleton and Rossi.^81^ The ascorbic acid contents in the leaves were calculated based on the titration of the Tillman standard solution of ascorbic acid at 0.02% according to the methodology of Norris.^82^

For nutritional analysis, the concentrations of nitrogen (N), phosphorus (P), carbon (C), silicon (Si), Si accumulation, and stoichiometry were evaluated. The C concentration was determined through the oxidation process using potassium dichromate in an acidic medium, followed by the titration of excess chromium (Cr^6+^). The N concentration was analyzed using the Kjeldahl method, which involves wet oxidation.^83^ The P concentration was evaluated by the nitric-perchloric acid digestion method, with subsequent measurement made by colorimetry using the ammonium metavanadate method.^83^ Finally, the silicon (Si) concentration was determined after alkaline digestion and colorimetric analysis with ammonium molybdate.^78,84^ The stoichiometric ratios of C/N, C/P, and C/Si were calculated from the nutrient concentrations in the plant tissues. Nutrient accumulation was determined by multiplying the dry mass by the respective concentrations of each nutrient.

#### Chitinolytic Activity of *Beauveria bassiana* Formulation

The evaluation of the Chitinase enzyme activity was performed using a qualitative assay based on an adapted methodology of Agrawal and Kotasthane.^85^ The presence of the enzyme is indicated by the color change of the culture media from yellow to pink and purple shades. The Pickering emulsions containing *B. bassiana* combined with the biogenic silicon nanoparticles (PE_ SiO_2__bio_Bb) and *B. bassiana* combined with the commercial silicon nanoparticles (PE_SiO_2__com_Bb) were analyzed, and the suspension of *B. bassiana* was used as a control for the Chitinase activity.

#### Evaluation of the Growth Kinetics of the Encapsulated Beauveria

The profile of fungal growth was evaluated according to the adapted methodology of Batista et al. and Bilesky-José et al.^86,2^ For this purpose, the Pickering emulsions incorporated with *B. bassiana* (Bb) were inoculated in PDA culture media by the addition of 10 μL of the samples in the center of the plates and exposed to ultraviolet (UV) radiation (12 h photoperiod). The nonencapsulated *B. bassiana* was also incubated and exposed to UV radiation as a comparison. Images were obtained in time intervals of 0, 1, 2, 4, 10, 12, 24, 48, 60, and 93 h and analyzed using ImageJ software. Fungal release was measured through the following equation:

$$\mathrm{growth}\left(t\right)=\frac{\mathrm{area}\left(t\right)-\mathrm{area}\left(t_{\mathrm{initial}}\right)}{\mathrm{area}\left(\mathrm{total}\right)-\mathrm{area}\left(t_{\mathrm{initial}}\right)}$$

where area­(*t*) corresponds to the fungal growth at time *t*, area­(*t*_initial_) corresponds to the start of mycelium growth outside the initial inoculation area, and area­(total) refers to the total Petri plate area.

#### Evaluation of the Phytotoxic Potential of the Microemulsions

The phytotoxic potential of silicon nanoparticles encapsulated in association with the *B. bassiana* fungus was evaluated using morphological characteristics and oxidative stress assays. The isolated nanoparticles and the fungus were also evaluated. Pre- and post-emergence treatments were applied to *Phaseolus vulgaris* (due to its rapid growth and its high economic importance as a crop) plants through seed treatment and foliar application, respectively. The employed treatments were (1) water (control); (2) pickering emulsion with the combined encapsulation of SiO_2_–NPs_bio and *B. bassiana* (10^8^ conidia mL^–1^) (PE_SiO_2__bio_Bb); (3) pickering emulsion with the combined encapsulation of SiO_2_–NPs_com and *B. bassiana* (10^8^ conidia mL^–1^) (PE_SiO_2__com_Bb); (4) 5% suspension of biogenic silicon nanoparticles (SiO_2_–NPs_bio_5%); and (5) fungal suspension of *B. bassiana* (10^8^ conidia mL^–1^) (Bb).

For the pre-emergent treatment, seeds were placed in contact with the samples and inserted into 10 cm diameter pots, with 5 pots per treatment and 5 seeds in each, irrigated daily, and kept under natural lighting and temperature conditions for 25 days, in a completely randomized design. Then, the plantlets were collected, and the roots were carefully washed to remove soil, followed by analysis of the morphological parameters, including shoot and root lengths, leaf area, and shoot and root fresh and dry masses.

For post-emergence treatment, planting was carried out under the same conditions mentioned above, but without prior seed treatment. After the emergence of the first trifoliate, the seedlings were treated with the samples by pulverization using approximately 0.35 g per leaf. The same parameters mentioned for the pre-emergent treatment were then analyzed.

The effects of the samples on the oxidative stress markers malondialdehyde (MDA) and hydrogen peroxide (H_2_O_2_) in pre- and postemergent treatments were also analyzed, according to methodologies adapted from Camejo et al. and Alexieva et al.^87,88^

#### Mortality Effects of the Nanoparticles and Formulations on *Spodoptera frugiperda*, *Spodoptera cosmioides*, *Chrysodeixis includens*, and *Rachiplusia nu* Caterpillars

The silicon biogenic nanoparticles and the formulation with the combined encapsulation of the nanoparticles and *Beauveria bassiana* were tested against *Spodoptera frugiperda, Spodoptera cosmioides*, *Chrysodeixis includens*, and *Rachiplusia nu* caterpillars, to investigate the potential of these nanomaterials for pest control. For this purpose, plastic pots with 5 mL capacity were prepared with artificial diet adapted from Greene et al. and supplemented with 75 μL of the samples.^89^ After diet drying, one caterpillar in the second instar was placed in each pot, resulting in 5 groups with 20 caterpillars per treatment. The caterpillars were then monitored daily for 7 days to analyze the lethal effects, and the number of dead individuals was recorded.

#### Statistical Analysis

Statistical studies were performed with two-way analysis of variance (ANOVA), followed by the Tukey test, with a significance level of *p* < 0.05, using GraphPad Prism 8.0 software. Kruskal–Wallis and Dunn tests were applied to the results of mortality effects on caterpillars, to verify differences between the data and the media groups, respectively.

## Supplementary Material



## Notes

Inoculated plates for postsynthesis monitoring, UV–vis spectra of the NPs, and emulsions studied (PDF)
