# Supplementary material for: Biogenic Silica Nanoparticles Enhance Beauveria bassiana Bioavailability and Efficacy for Sustainable Pest Control
Source: ACS Omega. 2026 May 13;11(20):29525–40. doi: 10.1021/acsomega.5c11436 (PMC13216996; doi:10.1021/acsomega.5c11436)
Supplement: Supplementary file 1 [file ao5c11436_si_001.pdf]

Supporting Information

Biogenic Silica Nanoparticles Enhance *Beauveria bassiana* Bioavailability and Efficacy for Sustainable Pest Control

Liliam Katsue Harada[1], Mariana Guilger-Casagrande[1], Tais Germano-Costa[1], Natália Bilesky-José[1], Ricardo Antonio Polanczyk[2], Kelly Cristina Gonçalves[2], Paulo Gonçalves da Silva[3], João Vitor Silva e Silva[3], Renato de Mello Prado[3], Leonardo F. Fraceto[4], Renata Lima[1]*

[1] Laboratory for Evaluation of the Bioactivity and Toxicology of Nanomaterials, University of Sorocaba (UNISO), Rod. Raposo Tavares km 92.5, Sorocaba, São Paulo, 18023-000, Brazil

[2] Laboratory of Microbial Control of Pests, São Paulo State University (UNESP), Via de Acesso Paulo Donato Castellane s/n, Jaboticabal, São Paulo, 14884-900, Brazil

[3] Laboratory of Plant Nutrition, Department of Agricultural Production Sciences (Soil and Fertilizer Sector), São Paulo State University (UNESP), Via de Acesso Paulo Donato Castellane s/n, Jaboticabal, São Paulo, 14884-900, Brazil

[4] Laboratory of Environmental Nanotechnology, São Paulo State University (UNESP), Av. Três de Março 511, Sorocaba, São Paulo, 18087-180, Brazil

Corresponding Author
Renata de Lima

Email: renata.lima@prof.uniso.br

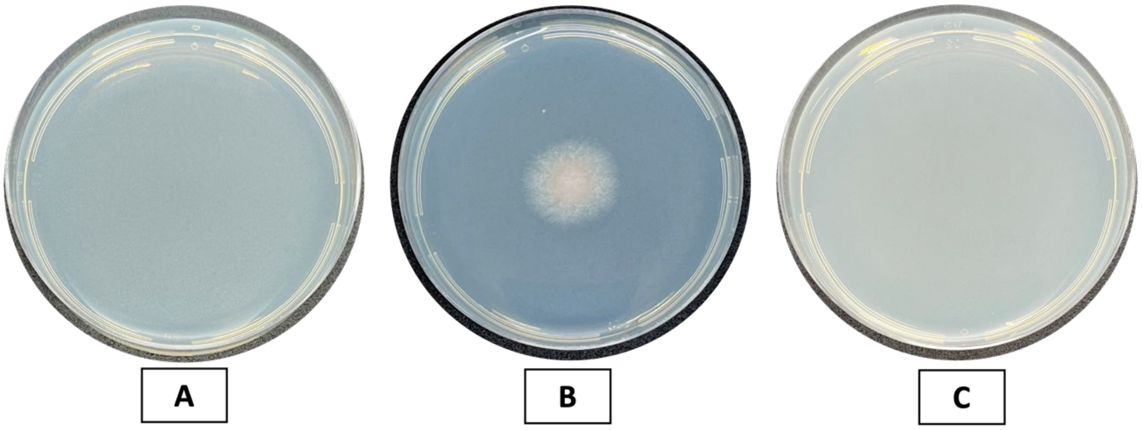

Figure S1 – Verification of the mycelial growth of the remaining *F. oxysporum* fungus on PDA: **A**) final suspension of biogenic silica nanoparticles; **B**) untreated biogenic silica nanoparticles suspension and **C**) ultrapure water.

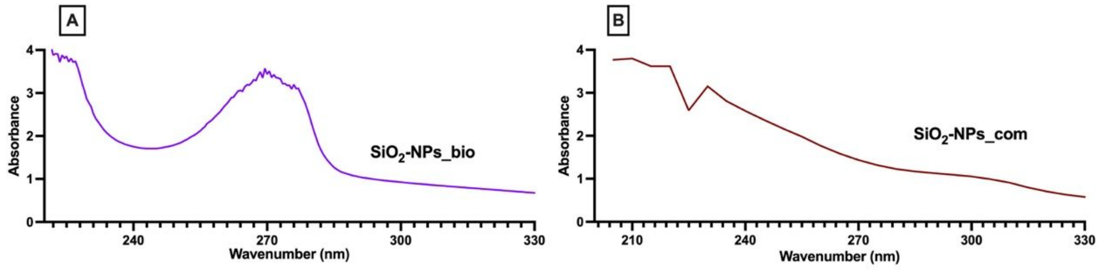

Figure S2 – Scanning spectra of (**A**) biogenic silica nanoparticles and (**B**) commercial silica nanoparticles.

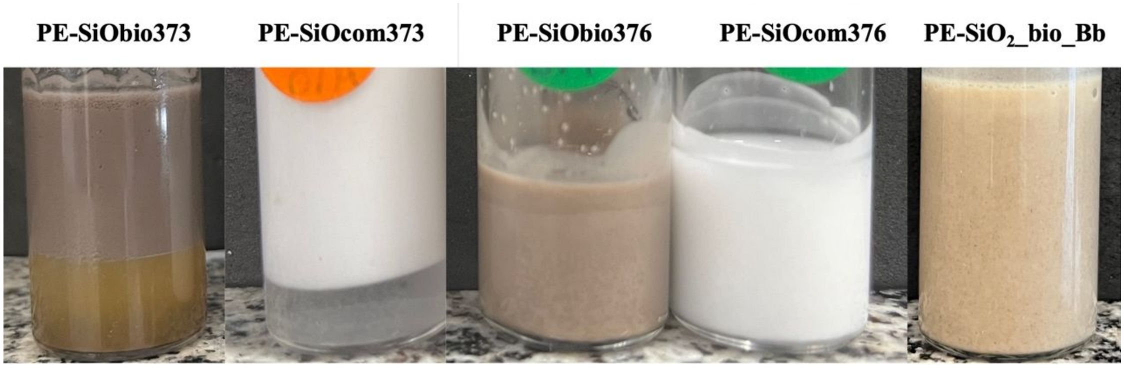

Figure S3 – Appearance of Pickering emulsions (oil-to-water ratio 3:7): PE-SiObio373 and PE-SiOcom373 stabilized with 3% functionalized nanoparticles, PE-SiObio376 and PE-SiOcom376 stabilized with 6% functionalized nanoparticles, biogenic and commercial, respectively, and the combination of PE-SiObio376 (Pickering emulsion) and *B. bassiana* (10^8 conidia. mL^{-1}) PE_SiO_2_bio_Bb.
